# Biomechanical Analysis of Hip Braces after Hip Arthroscopic Surgery for Femoroacetabular Impingement Syndrome: An Observational Study

**DOI:** 10.3390/biomimetics8020225

**Published:** 2023-05-26

**Authors:** Kai Hirata, Yoichi Murata, Akihisa Hatakeyama, Makoto Takahashi, Patrick M. Quinn, Soshi Uchida

**Affiliations:** 1 Research and Development Department, Nippon Sigmax Co., Ltd., Tokyo 160-0023, Japan; k_hirata@sigmax.co.jp; 2 Department of Orthopaedic Surgery, Wakamatsu Hospital of University of Occupational and Environmental Health, Kitakyushu 808-0024, Japan; yoichi0928111@gmail.com (Y.M.); pquinn0130@gmail.com (P.M.Q.); 3 Hatakeyama Orthopaedic Sports Clinic, Kitakyushu 808-0841, Japan; hatakeakihisa@gmail.com; 4 Department of Rehabilitation Medicine, Wakamatsu Hospital of University of Occupational and Environmental Health, Kitakyushu 808-0024, Japan; makonojou@yahoo.co.jp

**Keywords:** hip brace, biomechanics, femoroacetabular impingement, hip arthroscopy, capsular closure

## Abstract

Currently, hip braces are recommended and typically worn by femoroacetabular impingement (FAI) patients after hip arthroscopic surgery. However, there is currently a lack of literature regarding the biomechanical effectiveness of hip braces. The purpose of this study was to investigate the biomechanical effect of hip braces after hip arthroscopic surgery for FAI. Overall, 11 patients who underwent arthroscopic FAI correction and labral preservation surgery were included in the study. Standing-up and walking tasks in unbraced and braced conditions were performed at 3 weeks postoperatively. For the standing-up task, videotaped images of the hip’s sagittal plane were recorded while patients stood from a seated position. After each motion, the hip flexion-extension angle was calculated. For the walking task, acceleration of the greater trochanter was measured using a triaxial accelerometer. For the standing-up motion, the mean peak hip flexion angle was found to be significantly lower in the braced condition than in the unbraced condition. Furthermore, the mean peak acceleration of the greater trochanter was significantly lower in the braced condition than in the unbraced condition. Patients undergoing arthroscopic FAI correction surgery would benefit from usage of a hip brace in terms of protecting repaired tissues during early postoperative recovery.

## 1. Introduction

Femoroacetabular impingement syndrome (FAIS) was recognized as one of the most common sources of hip pain and dysfunction across the general population [1]. FAIS is defined as the abutment between a misshaped femoral head and acetabular rim, resulting in labral tears and cartilage damage, predisposing an individual to osteoarthritis of the hip. Some patients with FAIS also present with hip microinstability and/or posterior hip instability [2,3]. Recent studies proposed that hip instability is associated with impingement, as excessive acetabular anteversion was shown to result in anterior hip instability, and excessive acetabular retroversion resulted in anterior hip impingement or posterior hip instability many times [4]. Excessive femoral anteversion can result in anterior hip instability and posterior hip impingement, whereas excessive femoral retroversion can result in anterior hip impingement and posterior hip instability [5,6].

Over the years, hip arthroscopy became the gold standard procedure for assessing and treating patients with FAIS. In hip arthroscopic femoroacetabular impingement (FAI) correction surgery, the capsulotomy procedure allows for greater visualization of the hip joint and effective handling of instruments within the joint. Routine capsular closure was found to diminish the adverse effects of microinstability by restoring native capsular stabilization properties [2,7]. To preserve the positive results of capsular closure, it is necessary to restrict the extension and adduction of the hip during early postoperative rehabilitation to protect the capsular repair site. Additionally, hip flexion should be avoided to prevent stress at the labral repair site. Therefore, an appropriate dynamic hip brace is highly recommended post surgery, as it acts as a protector of the hip joint during the early phase of postoperative rehabilitation [8,9].

Several different hip braces were developed to protect and preserve the hip angle after hip arthroscopy surgery for FAI patients. In a kinematic assessment study on FAIS patients, Newcomb et al. analyzed 25 adults with FAIS undergoing a three-dimensional kinematic assessment of squat and stairlift movements using a motion capture system to investigate the effect of a hip brace on hip range of motion [10]. They found that hip brace utilization limited hip impingement during functional tasks but did not immediately reduce pain or improve patient-reported outcome measures for a young adult cohort with long-standing FAIS at four weeks post surgery. In addition, Michalik et al. measured the range of motion of the hip joint in study participants after they stood up from a chair wearing the brace, ultimately finding that the brace effectively limited hip range of motion [11]. Despite these results, few studies actively reported upon hip range of motion during hip brace usage in the early postoperative rehabilitation phase after hip arthroscopic surgery.

Diagnosing hip instability can be challenging for physicians, as there remains no definitive diagnostic test to utilize. In a previous study on hip instability, Maeyama et al. [12] conducted a gait experiment using a triaxial accelerometer for hip instability assessment in patients with developmental dysplasia of the hip (DDH). They found that hip instability in patients with DDH was significantly greater on the affected side than on the unaffected side. Additionally, Neira et al. [13] analyzed the acceleration of the greater trochanter using a triaxial accelerometer in subjects with and without hip instability. They found that participants with hip instability had a higher overall magnitude of acceleration than healthy controls. Patients with cam-type FAIS were shown to have greater hip instability than asymptomatic individuals with typical hip morphology [14]. However, there is currently a lack of literature on hip instability during hip brace usage after hip arthroscopic surgery in FAIS patients. Therefore, it is useful to evaluate the acceleration of the greater trochanter during hip brace usage in this patient cohort.

The purpose of this study was to investigate the biomechanical effectiveness of the hip brace after hip arthroscopy surgery for patients with FAIS. This study may provide clinical relevance for future postoperative rehabilitation protocols. We hypothesized that a hip brace would be effective in restricting the hip flexion-extension angle of the joint and reducing the acceleration of the greater trochanter.

## 2. Materials and Methods

### 2.1. Study Design

To investigate immediate hip brace effects, a within-participant design was used. To investigate effects of daily brace usage in patients with FAIS, an observational study design was used.

### 2.2. Setting

In this study, we recruited FAIS patients from May 2018 to December 2018 at the Wakamatsu Hospital of the University of Occupational and Environmental Health, Japan. The experiments were conducted at Wakamatsu Hospital of the University of Occupational and Environmental Health roughly 3 weeks after enrollment of each participant.

### 2.3. Participants

This study was approved by our institutional review board (approval number:H29-243). A total of 11 patients who underwent hip arthroscopic FAI correction (May 2018 to December 2018) and labral preservation (repair) for FAIS were enrolled in the study. Patients with functional, neurological, or morphological disorders affecting gait were excluded. Our inclusion criteria for symptomatic FAIS was based on the results of physical examinations and radiographs of symptomatic patients with FAIS. Inclusion criteria included hip pain for >3 months, restricted hip range of motion (ROM) (flexion < 105° and/or restricted internal rotation in flexion < 20°), and a positive impingement test result. Radiographic evidence of a cam deformity was defined as an alpha angle > 55° on at least one radiographic view, computed tomography, or magnetic resonance imaging [15,16]. Radiographic evidence of a pincer deformity was explored and defined as the presence of a crossover sign with a lateral center edge angle > 25°, a Tönnis angle < 0°, coxa profunda, or a positive ischial spine sign [15,16].

Radiographs of the 11 patients were assessed to determine the radiographic parameters in the diagnosis process of FAIS. We determined the lateral center edge angle, Tönnis angle, and alpha angle using the picture archiving and communication system (PACS). The lateral center edge angle was utilized to define lateral coverage of the acetabulum, while the Tönnis angle was utilized as a measure of acetabular inclination. The alpha angle was measured to identify cam-type impingement [17].

No patient in our study presented with a history of knee and ankle joint disease or injuries with normal radiographic findings. Every patient signed the approved informed consent form before participating and could walk independently 3 weeks prior to the study. At 3 weeks postoperatively, all participants performed standing-up and walking tasks in unbraced and braced conditions.

### 2.4. Introduction of the SU Hip Brace

**Figure 1:**
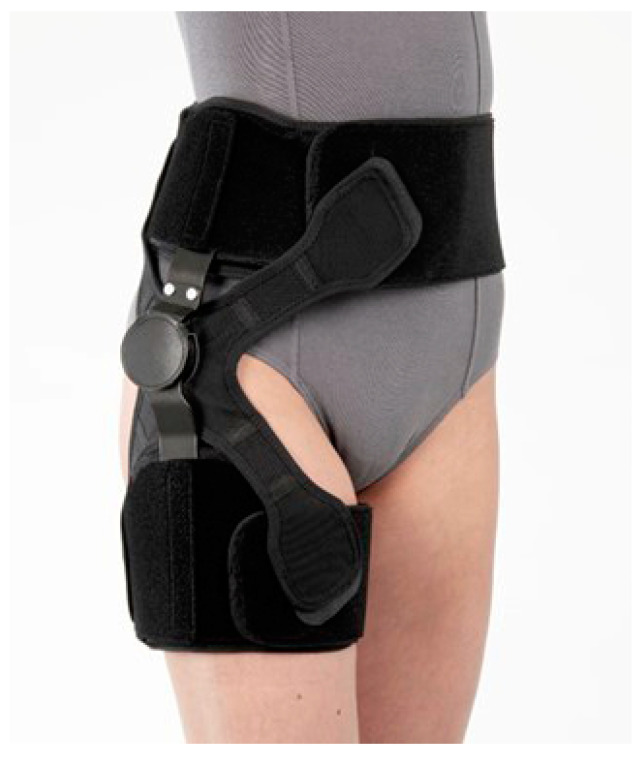
Appearance of the SU Hip Brace ( Nippon Sigmax Co., Ltd., Tokyo, Japan).

The test brace utilized in the current study was the SU Hip Brace (Nippon Sigmax Co., Ltd., Tokyo, Japan, Figure 1) and it weighed approximately 0.7 kg. It consisted of a hip hinge made of aluminum, a pelvis and thigh cuff made of fiber and reinforced by thermoplastic, and a hip strap made of fabric. Additionally, the hip hinge was flexible, allowing for individual patient fit. The pelvis and thigh cuff were deformed to precisely fit the pelvis and thigh of each patient. Furthermore, the angle adjustment block was able to restrict the range of the hip flexion-extension angle, while the hip strap reduced the instability of the joint.

While fitting the patient, the pelvis and thigh circumference of each patient were measured to choose the correct size and fit of the brace. A prosthetist and orthotist fitted the hip brace for each patient according to the brace’s instruction manual. During the fitting process, the hinge’s center was positioned such that it was located at the center of the hip joint. Straps around the pelvis and thigh were adapted to each patient’s pelvis and thigh circumference and adjusted for a firm fit. The center of the hip strap was positioned at the greater trochanter to push the greater trochanter inward. The average wearing period of the brace was approximately 3 weeks. Patients were instructed to apply the brace as taught as possible and wear it for as long as possible.

### 2.5. Surgery

The supine hip arthroscopic surgeries were performed on a traction table with a well-padded peroneal post by a single surgeon (senior surgeon, S.U.) with the patient under general or epidural anesthesia [18,19]. Anterolateral (ALP), mid-anterior (MAP), and proximal mid-anterior portals (PMAP) were established. Intra-articular inspection was first performed through two portals (ALP and MAP) using a 70° arthroscope with traction. The traction time was limited to <1.5 h for each patient to reduce the risk of neurovascular and skin injuries. An interportal capsulotomy was performed from the 12- to 3-o’clock perimetric position with an arthroscopic knife (Becton Dickinson, Franklin Lakes, NJ, USA) to facilitate arthroscopic visualization and instrument navigation.

Upon assessment, acetabular labral tears were found in all patients. The anterosuperior acetabular rim was exposed from the junction between the capsule and labrum with an elevator angulated at 10° and a radiofrequency probe, ensuring no disruption to the chondrolabral junction. Rim trimming was performed using a 5.5 mm motorized round burr when a pincer lesion was present and was synonymous with the acetabuloplasty procedure in the case of a pincer lesion and/or bone-bed preparation to promote labral healing. In all patients, the labrum was found to be unstable, and acetabuloplasty (rim trimming) was performed to refresh the bed and promote healing of the labral tissue. Additionally, anterior inferior iliac spine decompression (AIIS) was performed proximally for 1 to 1.5 cm with a motorized round-headed burr through the mid anterior portal if patients had symptomatic AIIS impingement. Labral repair with suture anchors was then performed, and microfracture was performed if patients possessed localized cartilage damage. After treatment of the central compartment, traction was released, and osteochondroplasty was performed when dynamic impingement confirmed that a cam lesion was present. Finally, shoelace capsular closure was performed with the hip at 30 to 40 degrees of flexion with the anterolateral portal as the viewing portal and mid-anterior or proximal mid-anterior portal as the working portal for all patients [6,19].

### 2.6. Standing-Up Task

**Figure 2:**
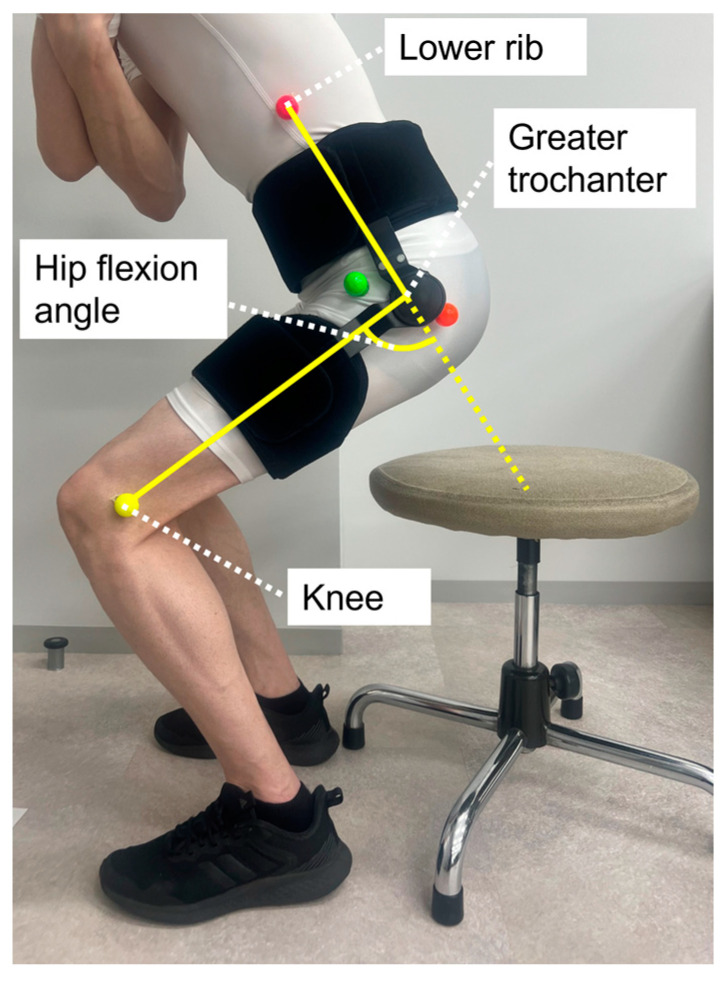
Image in the sagittal plane showing a participant performing the standing-up motion from a chair. The midpoints of the orange and green markers indicate the position of the greater trochanter.

The standing-up task was performed to investigate the restriction effect of the hip brace on the hip flexion angle. The reliability of evaluating the use of 2-dimensional motion analysis procedures that involve a video camera and software were demonstrated in a previous study [20]. During the task, each participant was instructed to repeat the motion of sitting to standing from a chair 5 times with their arms folded (Figure 2). The seat height was set such that the knee flexion angle was approximately 90°. The ROM of the hip brace was set between 0° and 75° by angle adjustment blocks. The standing-up motion was videotaped with a high-speed video camera (CASIO, EX-100pro, Tokyo, Japan) operating at 120 Hz for two-dimensional motion analysis. A high-speed video camera was placed approximately 5 m from the lateral side of the participant. The color markers were attached to the surface over the knee, greater trochanter, and lower rib on the surgical side using double-sided tape in the unbraced and braced conditions. Three-body landmarks, including the knee, greater trochanter, and lower rib, were digitized using MATLAB (Version 2019a, MathWorks, Natick, MA, USA), and 2-dimensional coordinate data for these landmarks were reconstructed. The hip flexion-extension angle was then calculated from the angles between the knee, greater trochanter, and lower rib in the sagittal plane. The hip flexion-extension angle in the neutral position was defined as 0°. The peak hip flexion angle was defined as the peak value of the hip flexion-extension angle, and the peak hip flexion angle was then compared between the braced and unbraced conditions.

### 2.7. Walking Task

**Figure 3:**
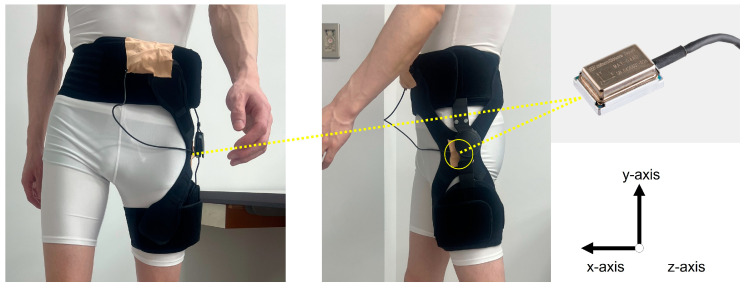
Coordinate system of the triaxial accelerometer during the walking task.

The walking task was performed to investigate the acceleration of the greater trochanter in the braced and unbraced conditions at 3 weeks postoperatively. The reliability of evaluating instability using accelerometry was demonstrated in a previous study [21]. As previous studies used an accelerometer to effectively measure hip instability during walking movements [12,13], we decided to use the same method. Each patient walked for approximately 15 m at their usual speed while wearing shoes a total of 5 times in each condition. To keep track of the walking speed for each trial, each patient practiced walking twice before the 5 measurement trials. An accelerometer (Microstone, MA3-04AD, Nagano, Japan) was used to record the triaxial acceleration during the walking motion, with the *x*-axis recording the anteroposterior direction, the *y*-axis recording the superior-inferior direction, and the *z*-axis recording the mediolateral direction (Figure 3). The sensor was attached to the surface over the greater trochanter on the surgical side using elastic tape in the unbraced condition and on the outside of the hip strap (on the greater trochanter) in the braced condition. The sampling rate was 1000 Hz. The mean peak values of the middle three gait cycles were used for data analysis. The overall magnitude of acceleration was calculated to evaluate instability of the hip joint using the following formula: $\left|a\right|=\sqrt{{ax}^{2}+{ay}^{2}+{az}^{2}}$. The peak overall acceleration was defined as the peak value of the overall magnitude of acceleration. The peak overall acceleration was compared between the braced and unbraced conditions.

### 2.8. Statistical Method

Comparisons of the peak flexion angle and peak overall acceleration between the braced and unbraced conditions were performed using a paired *t* test. A *p* value < 0.05 was considered statistically significant. All statistical analyses were performed using Excel 2016 (Microsoft, Redmond, DC, USA) on a laptop computer (HP, Palo Alto, CA, USA).

## 3. Results

### 3.1. Demographics

The patients’ backgrounds and demographic characteristics are shown in Table 1. The median age was 20 years (range, 15–40 years), and the cohort included ten male patients and one female patient. The median body mass index for all patients was 22.0 kg/m^2^ (range, 18.7–24.7 kg/m^2^), and the side of surgery included seven right and four left. During preoperative examination, the median lateral center edge angle was found to be 31° (range, 25°–36°), the median Tönnis angle was 4.8° (range, 1.1°–9.8°), and the median alpha angle was 69° (range, 61°–77°). Four patients presented with cam-type FAI, and seven patients presented with mixed-type FAI.

### 3.2. Standing-Up Task

**Figure 4:**
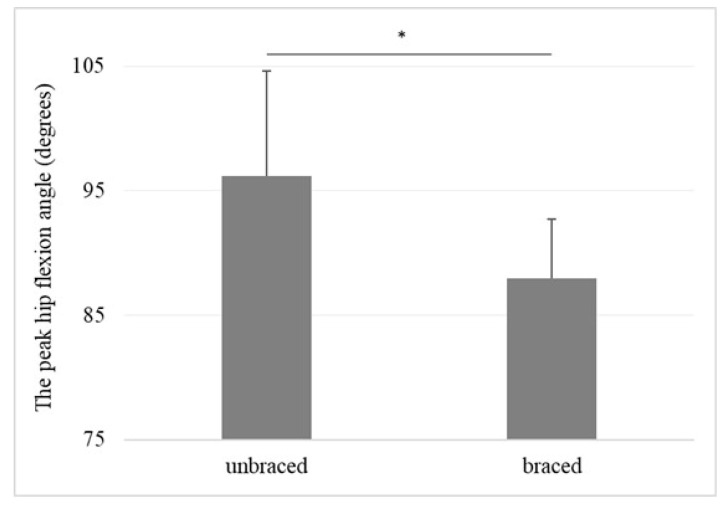
The mean peak hip flexion angle for the braced and unbraced conditions during the standing-up motion. Means and standard deviations of the means are presented. * *p* < 0.05. Braced: 88.0° (range 80.2–95.9°), Unbraced: 96.2° (range 84.4–106.4°).

**Figure 5:**
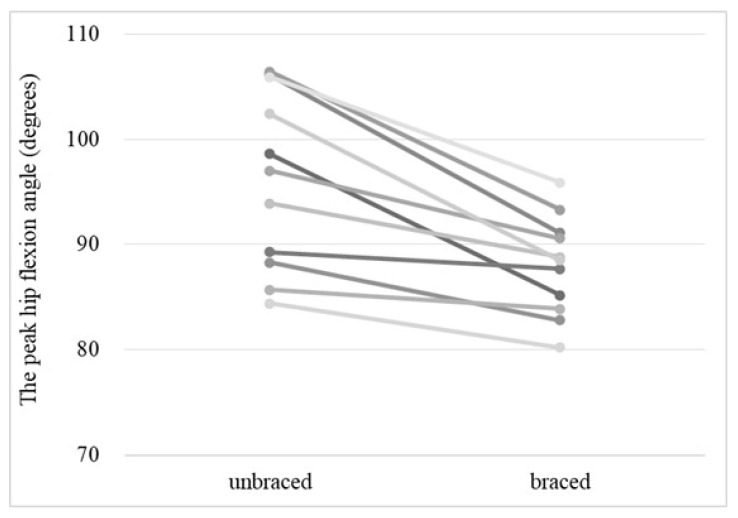
The individual peak hip flexion angle of the braced and unbraced conditions for each participant. Each line indicates the peak hip flexion angle of the braced and unbraced conditions for same participant.

The mean peak hip flexion angle of the braced and unbraced conditions is shown in Figure 4. The mean peak hip flexion angle during the standing-up motion was significantly lower in the braced condition than in the unbraced condition (88.0° [range 80.2–95.9°] vs. 96.2° [range 84.4–106.4°], *p* = 0.01). The individual peak hip flexion angles for the braced and unbraced conditions are shown in Figure 5.

### 3.3. Walking Task

**Figure 6:**
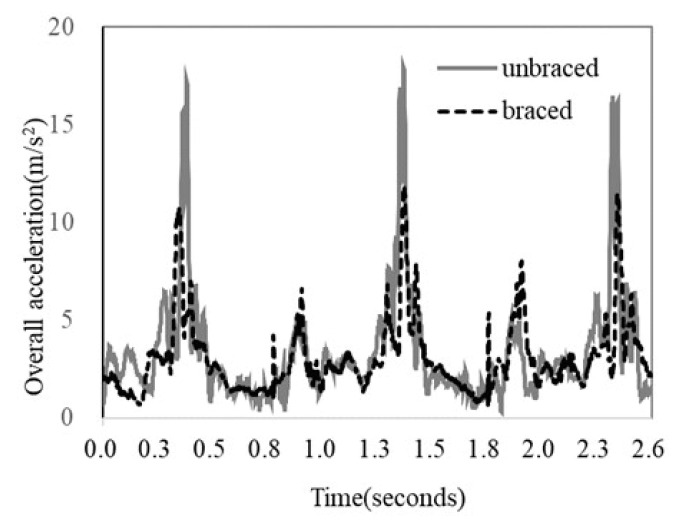
The overall acceleration of the greater trochanter during walking motion (typical pattern).

**Figure 7:**
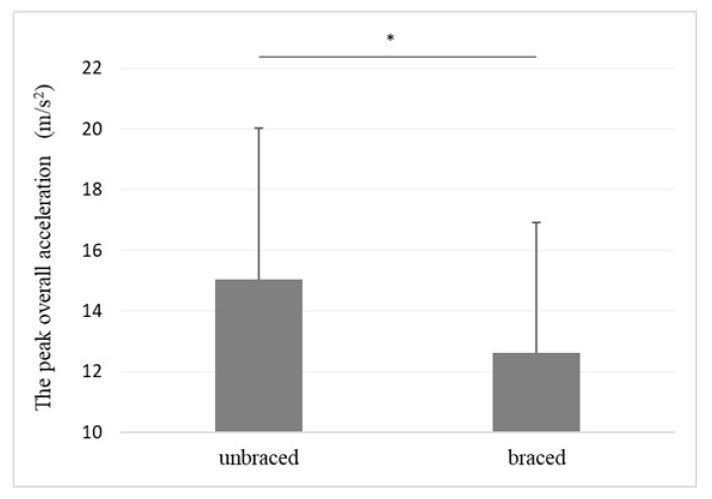
The mean peak overall acceleration in the braced and unbraced conditions. * *p* < 0.05. Braced: 12.6 m/s^2^ (range 7.4–19.5 m/s^2^), Unbraced: 15.0 m/s^2^ (range 8.2–21.0 m/s^2^).

**Figure 8:**
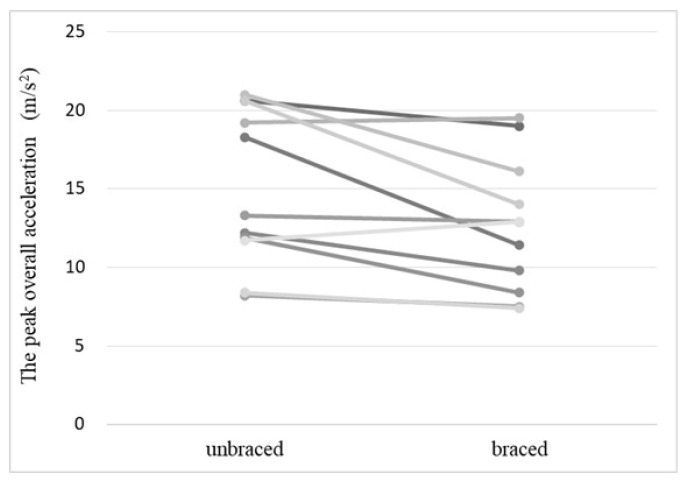
The individual peak overall acceleration in the braced and unbraced conditions for each participant. Each line indicates the peak overall acceleration of the braced and unbraced conditions for same participant.

The overall acceleration of the greater trochanter in the braced and unbraced conditions while walking (typical pattern) is shown in Figure 6. For the braced condition, the peak acceleration observed after foot contact was small. The mean peak acceleration of the greater trochanter of the braced and unbraced conditions is shown in Figure 7. The mean peak acceleration of the greater trochanter was significantly lower in the braced condition than in the unbraced condition (12.6 m/s^2^ [range 7.4–19.5 m/s^2^] vs. 15.0 m/s^2^ [range 8.2–21.0 m/s^2^], *p* = 0.02). The individual overall acceleration of the greater trochanter in the braced and unbraced conditions for every participant in the study is shown in Figure 8.

## 4. Discussion

There were two major findings in this study: (1) the peak hip flexion angle during the standing-up motion was significantly lower in the braced hip than in the unbraced hip, and (2) the peak acceleration of the greater trochanter during walking was significantly lower in the braced hip than in the unbraced hip.

Previous studies conducted a motion analysis to evaluate hip ROM in patients with FAIS who wore a hip brace. Newcomb et al. analyzed the squat, stair ascent, and stair descent motions of patients with FAIS using a motion capture system to investigate the effect of wearing a hip brace. They found that wearing the brace resulted in a slight decrease in flexion, internal rotation, and hip adduction [10]. Additionally, Michalik et al. measured the hip flexion-extension angle in healthy controls during the up and down motion from the seated position in a chair with a hip brace set at 70°, 90°, or no hip flexion limitation [11]. They reported that the use of a hip brace set up with flexion limitation did reduce hip ROM. It was considered that the hip brace could also restrict the hip flexion angle to reduce the risk of too much stress on the repaired labrum. However, the study found that measured hip flexion angles were greater than the settings of the hip brace allowed [11]. Similarly, in this study, the hip brace was set at 75° of flexion limitation using the angle adjustment block. However, the peak hip flexion angle during the standing-up motion was greater than the hip brace settings as well. Similar to the findings of the study by Michalik et al., the results of this study also suggest that to achieve a clinical limitation of 90° of hip flexion, the hip brace should be set at a smaller flexion limitation instead of a 90° flexion limitation.

Previous studies used the triaxial accelerometer to evaluate the dynamic instability of the hip during the walking motion. In a study on patients with hip dysplasia, Maeyama et al. reported a significantly higher overall acceleration of the hip on the affected side than on the asymptomatic contralateral side [12]. Additionally, Neira et al. reported a significantly higher overall acceleration in patients with atraumatic hip instability during walking than in asymptomatic controls [13]. Both reports concluded that the triaxial accelerometer is effective and reliable in evaluating the dynamic instability of the hip during walking movements. In agreement with the aforementioned evidence, our findings also showed a reduction in peak overall acceleration of the greater trochanter for the braced condition compared with the unbraced condition and a peak value of the greater trochanter acceleration for healthy and asymptomatic participants of 10.5 m/s^2^ during the walking task, which was quite similar to the peak value in the study by Neira et al. [13]. In this study, the peak value of the acceleration of the greater trochanter during the walking task was 12.6 m/s^2^ in the braced condition and 15.0 m/s^2^ in the unbraced condition, both of which were similar to the results of healthy and asymptomatic participants as reported by Neira et al. [13]. Thus, wearing a brace allows hip motions similar to those of healthy individuals. Furthermore, it is thought that the decrease in peak overall acceleration of the greater trochanter in hip brace patients is due to the strap of the hip brace during usage.

During hip arthroscopy, hip capsular release is commonly performed at the beginning of the procedure to improve visualization and accessibility of the scope and its instruments. Recent literature reported that capsular repair improves outcomes and accelerates return to sport rates after intra-articular lesion treatment [19,22]. Additionally, recent studies reported that revision arthroscopic hip capsular repair can improve clinical outcomes after failed initial hip arthroscopic surgery [6]. Murata et al. reported on the biomechanical evaluation of four capsular repair techniques for hip capsular management in a cadaveric study finding that the shoelace, double shoelace, and Quebec city slider techniques restored native stability (no significant difference from intact) in some but not all tests. Even though these techniques secured the sutures tightly, none perfectly restored native stability. Thus, we found a hip brace to be very useful for patients undergoing hip arthroscopic surgery in FAIS patients. In addition, we also found that performing only hip arthroscopy for some patients with generalized ligamentous laxity, such as patients with Ehlers–Danlos syndrome (EDS) or poor ligamentous tissue quality, is insufficient to restore adequate hip stability. Thus, we considered hip brace usage during postoperative management, especially for the capsular repair site, to be quite important [23].

Previous studies demonstrated the necessity of hip braces during the early phase of postoperative rehabilitation. Uchida et al. noted that it is necessary to avoid extension and external rotation to prevent stress on the closure site [24], while Safran et al. reported that labral tear damage is likely to occur when strong stress is generated by hip flexion, abduction, and internal rotation during this period [25]. Recently, Eiles et al. conducted a randomized control trial to examine the efficacy of a hip brace, looking at patient-reported outcome measures including the international hip outcome tool (iHot-33) and Copenhagen hip and Groin outcome scores (HAGOS). The authors found significant improvements in most HAGOS subscale scores favoring the hip brace group [26]. Domb et al. [27] found that the hip flexion of FAIS patients required limitation beyond 90° for the first three weeks after surgery to prevent labral retears and capsule rupture. In this study, the mean peak hip flexion angle of the braced condition was beyond 90°. However, the mean peak hip flexion angle in the unbraced condition was above 90°. From the aforementioned evidence, it was considered that the hip brace is useful for protecting the capsule closure site and labrum during postoperative rehabilitation because the peak hip flexion angle can be restricted.

This study had several limitations. First, it was difficult to evaluate hip adduction and internal rotation because of the patients’ postoperative status. To simplify things, we used a 2-dimensional motion analysis with a high-speed video camera instead of 3-dimensional motion analysis with a motion capture system. A triaxial accelerometer was attached to the skin over the greater trochanter to indirectly evaluate the movement of the femoral head. The transducer displays the acceleration of the limb during ambulation but is less useful in reliably detecting subtle subluxation of the femoral head and the presence of microinstability. The clinical relevance of wearing a hip brace remains elusive. Further investigations are necessary to evaluate the relationship between the clinical relevance and overall hip stability of using a hip brace. Second, this study was an experimental study performed without a control group (healthy hips). Furthermore, we did not collect the hip extension angle. It is necessary to evaluate the hip extension angle during hip brace usage after future hip arthroscopic FAI correction surgeries. Third, our sample size was relatively small, and most of the participants in this study were males because more males than females agreed to participate in this study. It is important to collect data from more female participants in the future. Fourth, we did not measure the velocity of the walking motion for each trial. To measure the walking speed for each trial, patients practiced walking twice before the five measurement trials. The velocity of each patient’s movement could have influenced acceleration of their body during the walking motion.

## 5. Conclusions

Hip braces can prevent excessive hip flexion angle and reduce the acceleration of the greater trochanter. Patients undergoing arthroscopic FAI correction surgery would benefit from the usage of a hip brace, in terms of protecting repaired tissues during early postoperative recovery.

## Figures and Tables

**Table 1:** Patients’ background.

Patient Demographics	Median (Range)
Age (years)	20 (15–40)
Sex	Men: 10, Women: 1
Body Mass Index (kg/m)	22.0 (18.7~24.7)
Side	Right: 7, Left: 4
Lateral center edge angle (degrees)	31 (25~36)
Tönnis angle (degrees)	4.8 (1.1~9.8)
Alpha angle (degrees)	69 (61–77)
Type of FAI	Combined: 7, Cam:4

Data are presented as median (range) values unless otherwise indicated.

## Data Availability

Not applicable.
